# Diagnostic accuracy and clinical utility of a new noninvasive index for hepatic steatosis in patients with hepatitis B virus infection

**DOI:** 10.1038/srep32875

**Published:** 2016-09-06

**Authors:** Zhiqiao Zhang, Gongsui Wang, Kaifu Kang, Guobiao Wu, Peng Wang

**Affiliations:** 1 Department of Infectious Diseases,The First People’s Hospital of Shunde, Shunde, Guangdong, China; 2 Department of Pathology, The First People’s Hospital of Shunde, Shunde, Guangdong, China

## Abstract

The aim of the present study was to construct a cost-effective noninvasive diagnostic index for prediction of hepatic steatosis in patients with hepatitis B virus(HBV) infection. From January 2011 to January 2015, a total of 364 consecutive subjects who underwent liver biopsies were enrolled. The Receiver-operating characteristic(ROC) curves and Obuchowski measure were constructed to evaluate the diagnostic accuracy of the new index. The AUROCs of steatosis index of patients with HBV infection (SIHBV) in predicting of steatosis were 0.929 (95% confidence interval:0.889–0.970, P < 0.05) in the model group and 0.855 (0.794–0.917, P < 0.05) in the validation group respectively. Comparisons of AUROCs demonstrated that SIHBV was significantly superior to Korean Score, fatty liver index (FLI), hepatic steatosis index (HSI), lipid accumulation product(LAP), and fatty liver disease (FLD) index for prediction of hepatic steatosis in model group and validation group(all P < 0.01). Especially for patients with hepatic steatosis percentage of 5.0–9.9% and 10.0–19.9%, SIHBV had a sensitivity of 63.6% and 79.2%, whereas it were 29.1% and 45.8% for Ultrasonography (all P < 0.05). In conclusion, as a cost-effective, simple, noninvasive, and readily available method, SIHBV may act as a massive screening tool before further examinations such as MRI, CT, transient elastography, or liver biopsy, especially for developing countries.

Hepatitis B virus (HBV) affects 350 million individuals in the world^1^. With improvement of living standard and lifestyle change, nonalcoholic fatty liver disease (NAFLD) has become a common liver disease, with a prevalence of 20–30% in the general population^2^. It has been reported that the incidence of hepatic steatosis ranged from 14.0% to 70.0% in patients with HBV infection^3^. Therefore, the coexistence of HBV infection and NAFLD has become a common phenomenon in liver disease. Remarkably, it has been reported that subjects with NAFLD had a higher risk of cardiovascular disease (CVD) than that without NAFLD^4^. Musso G *et al*. has reported that patients with NAFLD had an increased overall mortality for cardiovascular disease (28% of total deaths), extra-hepatic malignancies (25% of total deaths), and liver disease (13% of total deaths)^5^.

For patients with HBV infection, Jin *et al*. has reported that hepatic steatosis was an independent risk factor of failure for entecavir treatment by multivariate logistic regression at 24 week, 48 week and 96 week, indicating that hepatic steatosis was significantly associated with entecavir treatment failure^6^. Shi JP *et al*. has found that hepatic steatosis might be a influence factor associated with elevated alanine aminotransferase(ALT) levels in hepatitis B surface antigen(HBsAg)-positive chronic hepatitis B(CHB) patients with low HBV DNA loads, especially in patients treated with nucleoside analogs^7^. Similarly, Demir K has reported that nonalcoholic fatty liver disease was the most common cause of elevated ALT levels in Hepatitis B e Antigen(HBeAg) negative and HBV DNA negative patients^8^.

In clinical practice, clinicians often encounter newly diagnosed CHB patients with elevated ALT level and HBV-DNA positive status, or patients in the course of anti-viral therapy with abnormal liver function and low HBV-DNA load. Which is the major cause of elevated ALT level? CHB or NAFLD? For newly diagnosed CHB patients with elevated ALT level caused by NAFLD, it is not necessary to receive anti-viral therapy for the moment. For patients with abnormal liver function and low HBV DNA load in the course of anti-viral therapy, there is no need to adjust the anti-viral treatment if elevated ALT level was caused by NAFLD. Determination that whether fatty liver exists in CHB patients is very important for the final clinical decision. Therefore, CHB patients with elevated ALT level need a high cost-effective, noninvasive, sensitive, and convenient method for detection of NAFLD.

Liver biopsy is still the gold standard for assessing hepatic steatosis but is limited by invasiveness and sampling error^9^,^10^. Magnetic Resonance Imaging (MRI), Computed Tomography (CT), and transient elastography(TE) have a better diagnostic accuracy for detection of liver steatosis. However, these equipments are expensive and not readily available for most primary hospitals in developing countries. Furthermore, MRI, CT, and TE as a massive outpatient screening tool for hepatic steatosis do not comply with the principle of cost-effectiveness.

Most primary hospitals select Ultrasonography(US) as basic screening tool for detection of NAFLD in consideration of clinical maneuverability and cost-effectiveness. From the point of view of cost-effectiveness, US is a high cost-effective noninvasive method for detection of fatty liver. However, US is limited by low sensitivity for mild steatosis and inability to differentiate mild fibrosis from steatosis^11^. More importantly, Ryan CK *et al*. has reported that US could only detect 55% and 72% of patients with hepatic steatosis percentage of 10–19% and 20–29%, respectively;meanwhile, US could not detect hepatic steatosis while hepatic steatosis presented in less than 10% of hepatocytes^12^. The results of these previous studies suggested that US was limited in patients with mild hepatic steatosis.

Several noninvasive diagnostic indexes have been established to predict hepatic steatosis in general population. In 2005, Poynard *et al*. has conducted a noninvasive diagnostic index named steatoTest, showing an AUROC of 0.790 (SE 0.03) for hepatic steatosis ≥5% by biopsy in the training group^13^. Bedogni *et al*. has established Fatty Liver Index (FLI) in a population of 496 subjects with hepatic steatosis diagnosed by US in 2006, with an AUROC of 0.840 (95% CI 0.810–0.870)^14^. In 2010, Bedogni *et al*. has constructed another diagnostic index named lipid accumulation product (LAP), with an AUROC of 0.80 (95% CI 0.760–0.840)15. However, these studies were performed in general population, which may not be suitable for patients with HBV infection.

Therefore, we performed this retrospective study to construct a high cost-effective and noninvasive diagnostic index for prediction of hepatic steatosis in patients with HBV infection. To assess the diagnostic accuracy of this new index, we compared it with US and five other noninvasive indexes for detection of liver steatosis in CHB patients.

## Patients and Methods

### Patients

This study included 431consecutive patients who had been diagnosed with HBV infection and had undergone liver biopsies in Department of Infectious Diseases of The Shunde First People’s Hospital between January 2011 and January 2015. The Patients were enrolled based on the following criteria: chronic hepatitis B(CHB) defined as hepatitis B surface antigen (HBsAg) positivity for more than 6 months; HBV-DNA >10^3^ copies/ml. The exclusion criteria were as follows: liver cancer(n = 5);co-infection with hepatitis C virus, hepatitis D virus or human immunodeficiency virus(n = 8); autoimmune liver diseases suah as autoimmune hepatitis, primary biliary cirrhosis, and primary sclerosing cholangitis(n = 3); alcohol ingestion >20 g/day (n = 15); hereditary and metabolic liver diseases such as Wilson’s disease, hemochromatosis, and α-1-antitrypsin deficiency(n = 2). The patients with missing data in terms of BMI, TG, LDL, UA, and HGB were ruled out(n = 34).

Therefore, a total of 364 patients (298 males and 66 females) were recruited into the present study finally. There were no significant differences in terms of demographic and clinical parameters between patients included and excluded (data not shown). The written informed consent were obtained from patients before inclusion. This study was approved by the ethics committee of The First People’s Hospital of Shunde. All clinical investigations were conducted according to the principles of Declaration of Helsinki.

### Biochemical examination and noninvasive diagnostic indexes

All patients systematically underwent complete biochemical workups, ultrasonography, and liver biopsy within 2 days. Blood samples of the subjects were obtained before liver biopsy. Biochemical tests were performed by commercial assays in our hospital laboratory. The serum HBV-DNA load was detected using a Real-Time polymerase chain reaction (PCR) System (ABI7700; Applied Shenzhen city Daeran Biological Engineering Co Ltd, Shenzhen, Guangdong, CHN).

Waist circumference was measured at the midway point between the costal margin and the iliac crest. Body mass index (BMI) was calculated as weight (kg)/height (m)^2^.

The Korean Score, fatty liver index (FLI), hepatic steatosis index (HSI), lipid accumulation product(LAP), and fatty liver disease (FLD) index were calculated according to the formulas reported in the original articles^13^,^14^,^15^,^16^,^17^.

Korean Score was calculate according to the following parameters:ALT/AST ratio > 1.5, 1 point; γ-GTP (IU/L) > 50, 1 point; Triglyceride (mg/dL) > 150, 1 point; 23 kg/m^2^ ≤ BMI < 25 kg/m^2^, 1 point; 25 kg/m^2^ ≤ BMI, 2 points.


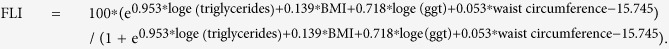



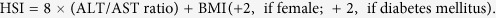



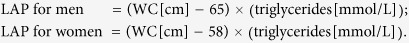



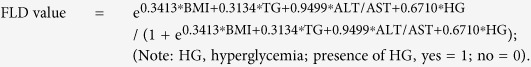


We performed this study according to the STARD recommendation for the optimal quality in reporting diagnostic accuracy.

### Liver biopsy

**Figure 1:**
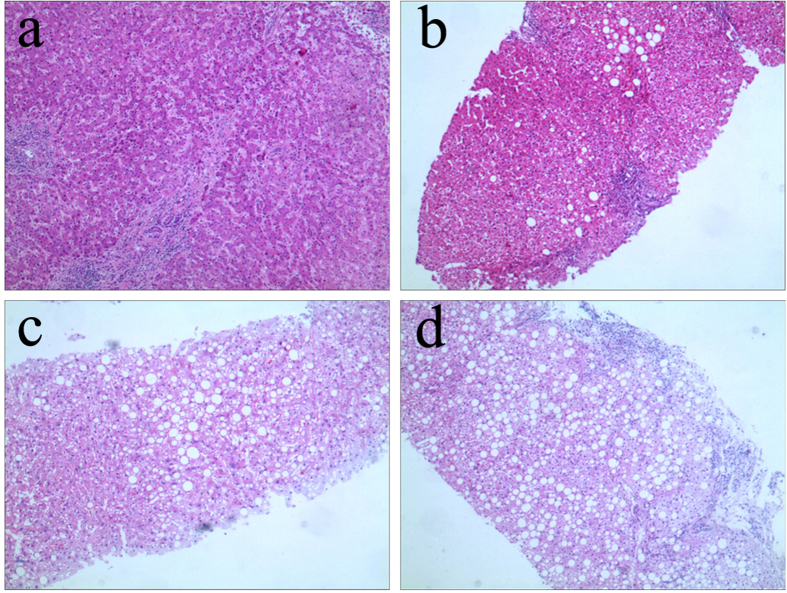
Pathological characteristics of hepatic steatosis in patients with hepatitis B virus infection (HE staining). (**a**) None steatosis (×200). (**b**) Mild steatosis (×200). (**c**) Moderate steatosis(×200). (**d**) Severe steatosis (×200).

We used Menghini’s method of one second rapid vacuum aspiration(16G biopsy Menghini’s needle, ShangHai) to get liver tissue. A minimum length of 2.0 cm with at least 11 portal tracts was required for qualified liver specimens. The specimens were fixed, paraffin-embedded, and stained with haematoxylin and eosin (HE). Histological grading of necro-inflammation (G0-G4) and staging of the liver fibrosis (S0-S4) were carried out according to Scheuer’s classification^18^ by one experienced pathologist blinded to the clinical data. Hepatic steatosis was graded according to the percentage of hepatocytes affected (Fig. 1): none(<5%), mild steatosis (5–32%), moderate steatosis (33–65%), and severe steatosis (≥66%)^19^. Steatosis group was defined as steatosis ≥5% of hepatocytes and non-steatosis group was defined as steatosis <5% of hepatocytes.

### Statistical analysis

Continuous data were expressed as mean ± SD or median (quartile range) depending on the normality of the data. Categorical variables were expressed as proportions. Continuous variables were compared by one-way ANOVA analysis of variance or Kruskal-Wallis H test, depending on the normality of the data; Nominal or ordinal variables were compared by Kruskal-Wallis H test. All variables that significantly associated with hepatic steatosis in univariate logistic regression analyses were included in forward stepwise multivariate logistic regression analysis to conduct a predictive index for hepatic steatosis. The area under the receiver operator characteristic curve(AUROC) were calculated to evaluate the diagnostic accuracy of new index. The AUROC values of these indexes were compared by DeLong’s test^20^. The Obuchowski measure method was used to take into account of all pair-wise comparisons between different stages of hepatic steatosis to reduce the spectrum effect and risk of multiple testing^21^,^22^,^23^. The Obuchowski measure can be interpreted as the probability that the noninvasive index will correctly rank two randomly chosen patient samples from different grades according to the weighting scheme, with a penalty for misclassifying patients^21^,^22^,^23^. The results of Obuchowski measure may be not equivalent to the original area under ROC curve for the reason of that the Obuchowski measures are weighted according to the distance between stages of hepatic steatosis. Statistical analyses were performed using SPSS 19.0 (SPSS Inc., Chicago, IL). *P* < 0.05 was considered statistically significant.

## Results

### Baseline characteristics of subjects in model group and validation group

A total of 364 patients were included in the study, 118 (32.4%) of whom were diagnosed with hepatic steatosis. All patients included in the present study were randomly divided into two groups: model group (n = 182) and validation group (n = 182). The baseline characteristics of subjects in model group and validation group were summarized in Table 1.

### Selection of variables for predicting hepatic steatosis by univariate and multivariate logistic regression analyses

All variables that significantly associated with hepatic steatosis in univariate logistic regression analyses were included in forward stepwise multivariate logistic regression analysis to conduct a predictive index for hepatic steatosis. The results of logistic analyses were summarized in Table 2. BMI, hemoglobin, age, TG and SUA were included in the diagnostic indexl for hepatic steatosis in patients with HBV infection finally.

According to the results of multivariate logistic regression analysis, we constructed a new diagnostic indexl named steatosis index of patients with HBV infection (SIHBV):


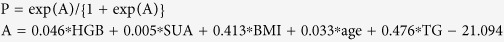


### Diagnostic accuracy of SIHBV for prediction of hepatic steatosis in model group

**Figure 2:**
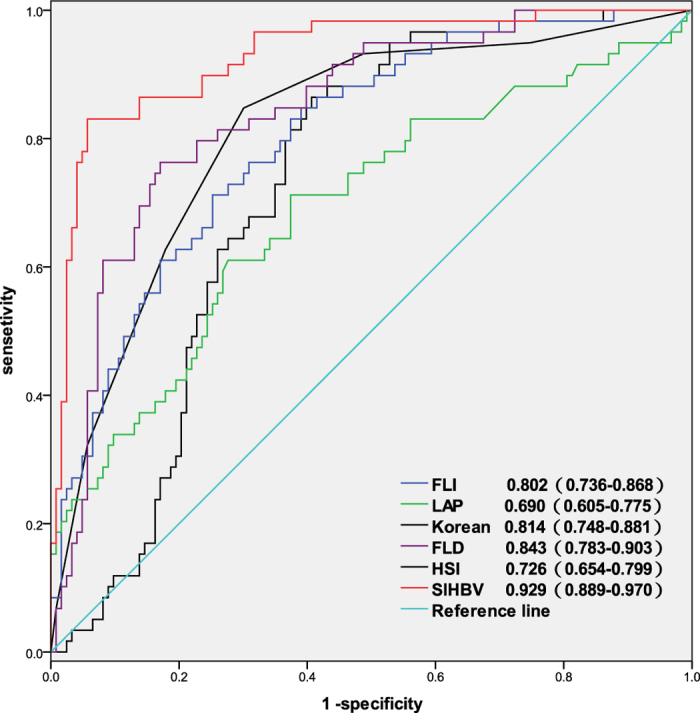
AUROCs of SIHBV, FLI, LAP, HSI, FLD, and Korean Score for prediction of hepatic steasosis in model group. Data in the figure were presented as AUROC (95% CI).

The AUROC of SIHBV was calculated to assess the diagnostic accuracy for prediction of hepatic steatosis (Fig. 2). The AUROC of SIHBV in predicting of hepatic steatosis was 0.929 (95% CI:0.889–0.970, *P* < 0.05), which was higher than that of Korean, LAP, HSI, FLI, and FLD. Comparisons of AUROCs using method suggested by Delong^21^ demonstrated that SIHBV was significantly superior to FLI, LAP, HSI, FLD, and Korean Score for prediction of hepatic steatosis in model group (all P < 0.001).

### Comparisons of AUROCs of six indexes in predicting hepatic steatosis in validation group

**Figure 3:**
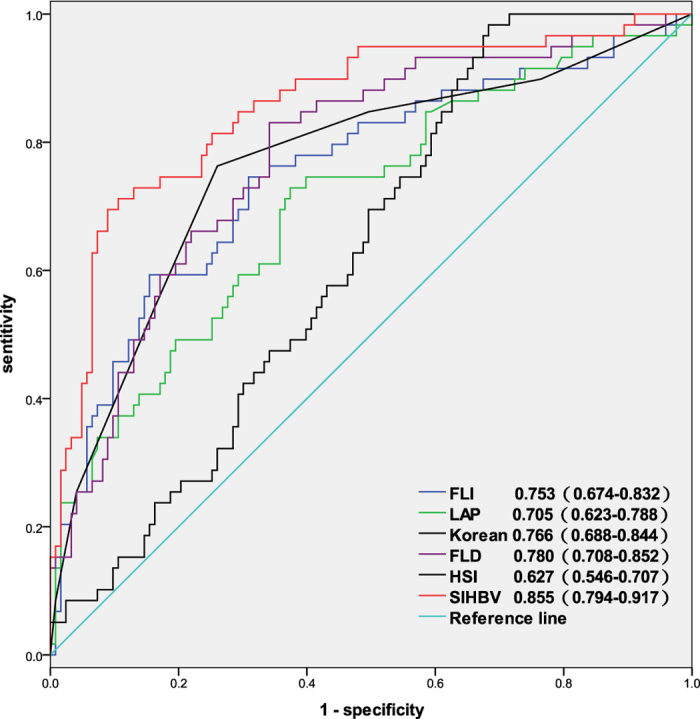
AUROCs of SIHBV, FLI, LAP, HSI, FLD, and Korean Score for prediction of hepatic steasosis in validation group.

The AUROCs of SIHBV, FLI, LAP, HSI, FLD, and Korean Score (Fig. 3) in predicting hepatic steatosis were 0.855 (0.794–0.917), 0.753 (0.674–0.832), 0.705 (0.623–0.788), 0.627 (0.546–0.707), 0.780 (0.708–0.852), and 0.766 (0.688–0.844), respectively. Similarly, Comparisons of AUROCs also indicated that SIHBV was significantly superior to FLI, LAP, HSI, FLD, and Korean Score for prediction of hepatic steatosis in validation group(all *P* < 0.01).

### Comparisons of the AUROCs of six noninvasive indexes by Obuchowski measure

The spectrum effect reflects the inherent variation of performance of diagnostic models in different population. Considering of spectrum effect caused by different distributions of hepatic steatosis stage, the results of comparisons of AUROCs by Delong test need further confirmation.

The Obuchowski method can fully take into account of the spectrum effect and the ordinal scale system^21^,^22^,^23^. The Obuchowski measure was a weighted average of AUROCs for prediction of hepatic steatosis between two of the total four stages in the present study. Each pair-wise comparison was weighted by the numbers of patients. The distance between two stages of hepatic steatosis was taken into account. A penalty function proportional to the difference between hepatic steatosis stages was defined as follows: the penalty function was 0.5 when the difference between stages was 1, 0.75 when the difference was 2, and 1 when the difference was 3.

The overall diagnostic accuracy of SIHBV (Obuchowski measure = 0.924)for hepatic steatosis was significantly higher than that of FLD (Obuchowski measure = 0.879), Korean (Obuchowski measure = 0.867), FLI (Obuchowski measure = 0.860), LAP (Obuchowski measure = 0.821), and HSI (Obuchowski measure = 0.811).

### Explore of clinical utility of SIHBV for different thresholds of hepatic steatosis

**Figure 4:**
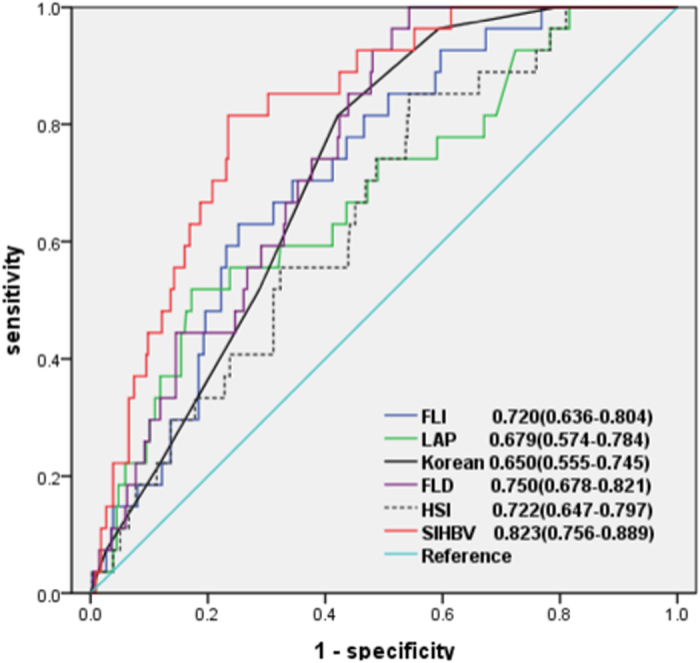
AUROCs of SIHBV, FLI, LAP, HSI, FLD, and Korean Score for prediction of hepatic steasosis with a threshold of 33%.

We further explored the clinical utility of SIHBV according to different thresholds of hepatic steatosis. For a threshold of 33% of hepatic steatosis, 27 and 337 patients were defined as with or without hepatic steatosis. The AUROCs of SIHBV, FLI, LAP, HSI, FLD, and Korean Score (Fig. 4) in predicting hepatic steatosis were 0.823 (0.756–0.889), 0.720 (0.636–0.804), 0.679 (0.574–0.784), 0.650 (0.555–0.745), 0.750 (0.678–0.821), and 0.722 (0.647–0.797), respectively. Comparisons of AUROCs by DeLong’s test indicated that SIHBV was significantly superior to FLI, LAP, HSI, FLD, and Korean Score for prediction of hepatic steatosis for a threshold of 33% (all *P* < 0.05).

**Figure 5:**
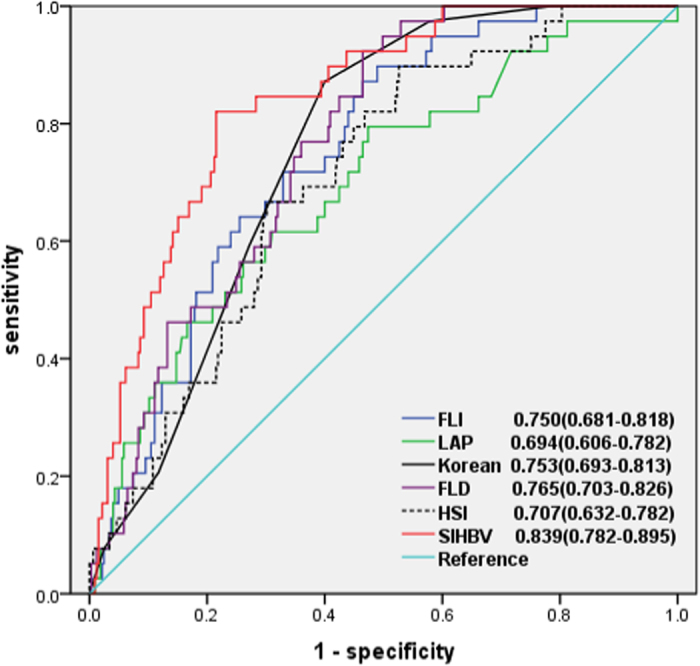
AUROCs of SIHBV, FLI, LAP, HSI, FLD, and Korean Score for prediction of hepatic steasosis with a threshold of 20%.

For a threshold of 20%, 39 and 325 patients were defined as with or without hepatic steatosis. The AUROCs of SIHBV, FLI, LAP, HSI, FLD, and Korean Score (Fig. 5) in predicting hepatic steatosis were 0.839 (0.782–0.895), 0.750 (0.681–0.818), 0.694 (0.606–0.782), 0.707 (0.632–0.782), 0.765 (0.703–0.826), and 0.753 (0.693–0.813), respectively. Comparisons of AUROCs by DeLong’s test showed that SIHBV was significantly superior to FLI, LAP, HSI, FLD, and Korean Score for prediction of hepatic steatosis for a threshold of 20% (all *P* < 0.01).

### Comparisons of the diagnostic accuracy between US and SIHBV in different steatosis stages

In all 118 patients with steatosis diagnosed by liver biopsy, US only detected 29.1, 45.8, 58.3, and 81.5% of patients with hepatic steatosis percentage of 5.0–9.9%, 10.0–19.9%, 20.0–32.9, and 33.0–100.0%, respectively (Table 3). The positive rate of fatty liver detected by SIHBV was 63.6, 79.2, 83.3, and 81.5% for patients with hepatic steatosis percentage of 5.0–9.9%, 10.0–19.9%, 20.0–32.9%, and 33.0–100.0% (Table 3). The comparison results of Chi square test indicated that SIHBV was significantly superior to US for hepatic steatosis percentage of 5.0–9.9% and 10.0–19.9% (*P* = 0.001 and *P* = 0.018). For hepatic steatosis percentage of 20.0–29.9%, sensitivity of SIHBV was higher than that of US, although the difference was not statistically significant(P > 0.05). For hepatic steatosis percentage of 33.0%–100.0%, SIHBV had the same sensitivity as US (P > 0.05). The comparison results of Chi square test also indicated that the total correct rate of SIHBV was significantly superior to US(P = 0.027) Table 3.

### Clinical utility and practical value of SIHBV for prediction of hepatic steatosis

To explore the clinical utility of SIHBV for prediction of hepatic steatosis, the optimal Cut-off values were determined according to positive likelihood ratio (PLR) ≈ 10.0 for confirming diagnosis of hepatic steatosis and negative likelihood ratio (NLR) ≈0.1 for excluding diagnosis of hepatic steatosis^24^.

**Figure 6:**
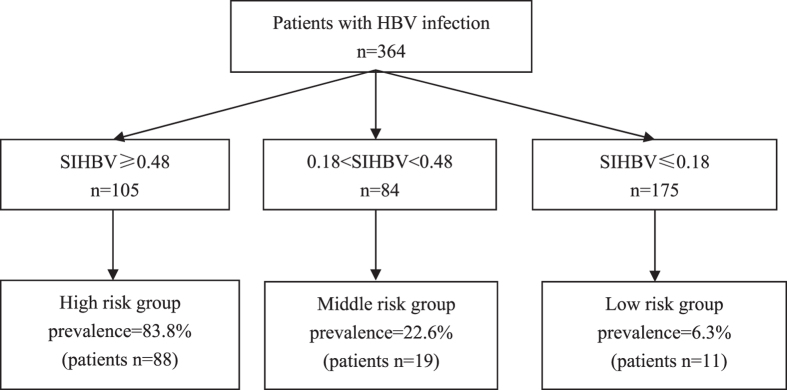
Risk stratification chart of SIHBV for prediction of hepatic steatosis.

Among the 364 patients included in the study, 175 (48.1%) patients had a value of SIHBV less than or equal to 0.18 and 105 (28.8%) patients had a value of SIHBV higher than or equal to 0.48 (Fig. 6). The cut-off value of 0.18 showed a LR- of 0.14, a NPV of 93.7%, and a sensitivity of 90.7%. The patients with value of SIHBV less than or equal to 0.18 could be defined as low risk group with a prevalence rate of 6.3% and a correct diagnostic rate of 93.7%. The cut-off value of 0.48 showed a LR+ of 10.8, a PPV of 83.8%, and a specificity of 93.1%. The patients with value of SIHBV higher than or equal to 0.48 could be defined as high risk group with a prevalence rate of 83.8% and a misdiagnosis rate of 16.2%.

## Discussion

We constructed a new diagnostic index for prediction of hepatic steatosis in patients with HBV infection, consisting of BMI, hemoglobin, age, TG and SUA. The AUROC of SIHBV in predicting hepatic steatosis were 0.929 (0.889–0.970) in the model group and 0.855 (0.794–0.917) in the validation group. Comparisons of AUROCs indicated that SIHBV was significantly superior to US, FLI, LAP, HSI, FLD, and Korean Score for prediction of hepatic steatosis in model group and validation group (all *P* < 0.01). Interestingly, the further comparison results of Chi square test indicated that SIHBV was significantly superior to US for hepatic steatosis percentage of 5.0–9.9% and 10.0–19.9%, demonstrating that SIHBV was valuable for detection of mild hepatic steatosis and could help clinicians to find mild hepatic steatosis in patients with HBV infection. With a lower value of 0.18 and a higher value of 0.48, the patients were divided into three groups with different prevalence risk of hepatic steatosis, facilitating clinicians assess the risk of NAFLD and distinct that whether elevated ALT level was caused by NAFLD. Comparisons of Obuchowski measure confirmed that SIHBV was superior to other five noninvasive indexes for hepatic steatosis.

SIHBV consisted of BMI, hemoglobin, age, TG, and SUA. BMI and TG were found to be independent risk factors for hepatic steatosis in previous studies^25^,^26^. Jin *et al*. has found that serum uric acid was an independent risk factor for hepatic steatosis^6^. Trak-Smayra *et al*. has showed that serum free hemoglobin subunits were correlated with severity of liver lesions in NAFLD and might serve as a biomarker for the disease^27^. Xu *et al*. has reported that serum hemoglobin concentration was significantly associated with NAFLD and the prevalence rate of NAFLD increased with progressively higher serum hemoglobin concentrations^28^. Yu *et al*. has found that subjects with higher baseline hemoglobin level were associated with higher incidence of NAFLD and serum hemoglobin might has significant predictive value for NAFLD through a prospective analysis^29^. In agreement with these studies above, multivariate logistic regression analysis in the present study also demonstratede that BMI, hemoglobin, age, TG and SUC were independent influence factors of hepatic steatosis.

Considering the invasiveness and cost, liver biopsy is not suitable to be a routine tool for massive screening of hepatic steatosis. MRI, CT, and TE are too expensive to be a screening tool for hepatic steatosis and not readily available in most primary hospitals. US is a simple noninvasive method, which is suitable for detection of fatty liver. However, we must consider that the low sensitivity for mild steatosis and the accumulated cost of repeated examinations in the treatment. Therefore, we consider that SIHBV is a good candidate for massive outpatient screening tool for hepatic steatosis as the following reasons: First, results of the present study has demonstrated that SIHBV was significantly superior to US, FLI, LAP, HSI, FLD, and Korean Score for prediction of hepatic steatosis in model group and validation group. Second, SIHBV also had a better sensitivity for detection of mild hepatic steatosis even in patients with hepatic steatosis percentage of 5.0%–9.9% and 10.0–19.9%. Third, all informations of BMI, hemoglobin, age, TG, and SUA could be freely obtained from the results of routine examinations, meaning that SIHBV was actually a free method in the clinical practice. Complying with the principle of cost-effectiveness, SIHBV, as a simple, noninvasive, free, and readily available method, is a good choice before further examinations such as MRI, CT, TE, or liver biopsy.

The threshold of hepatic steatosis in the present study was defined as 5%. We further explored the diagnostic accuracy of SIHBV in different thresholds such as 33 and 20%. The results demonstrated that SIHBV was significantly superior to other five indexes for thresholds of 33 and 20% by DeLong’s test. However, for thresholds of 33 and 20%, there were only 27 and 39 patients which were defined as with hepatic steatosis. Therefore, a larger cohort is needed to prove and validate the effectiveness of SIHBV for moderate-severe steatosis.

In clinical practice, patients with CHB may benefit from SIHBV in several aspects as follows: First, a substantial part of patients with CHB will avoid unnecessary liver biopsy. The patients with SIHBV ≤ 0.18 can be excluded diagnosis of hepatic steatosis with a correct diagnostic rate of 93.7% and a LR− of 0.14. The patients with SIHBV ≥ 0.48 can be diagnosed as hepatic steatosis with a specificity of 93.1% and a LR+ of 10.8. With cut-off values of 0.18 and 0.48, 280 (76.9%) of 364 patients can be free from liver biopsy with a overall correct rate of 90.0% for diagnosis of hepatic steatosis. Second, patients with CHB can save medical expenditures in the course of treatment by replacing US with SIHBV, which is actually a free method for BMI, hemoglobin, age, TG, and SUA can be freely obtained from routine examinations. Most importantly, SIHBV can help doctors determine the real cause of elevated ALT in the course of treatment or selection of suitable patients for antiviral therapy, especially hepatic steatosis percentage is less than 20%.

It has been reported that the prevalence of NAFLD and non-alcoholic steato-hepatitis (NASH) were 76 and 56% in patients with Type 2 Diabetes Mellitus(T2DM) and normal plasma aminotransferase levels, indicating that NAFLD and NASH were common in patients with T2DM^30^. Subjects with T2DM had a higher prevalence of severe NAFLD than those without T2DM and increased hepatic steatosis was significantly associated with the presence of T2DM^31^. Due to the high prevalence of obesity and type 2 diabetes mellitus (T2DM), the coexistence of HBV infection and NAFLD has become a common phenomenon in the world. Soverini *et al*. has found that patients with HBV and/or HCV infections had higher liver enzyme levels in comparison with virus-negative patients (*P* < 0.0001), whereas the prevalence of the metabolic syndrome was similar in this 2 groups^32^. The elevated liver enzymes might be frequently disregarded in diabetes Units and ascribed to metabolic syndrome, thus excluding T2DM patients from specific disease-modifying antiviral treatment for hepatitis. For patients with T2DM and HBV infection, the identification of the major cause of elevated liver enzyme levels was important for the clinical treatments. Therefore, SIHBV is particularly useful for patients with T2DM and HBV infection.

The present study has several strengths: First, SIHBV consists of readily-available laboratory parameters which can be freely obtained from routine examinations, so SIHBV is easy to be implement in the primary hospital. Second, the prevalence and stages of hepatic steatosis were different in various studies, which should be taken into account for assessment of diagnostic accuracy of noninvasive indexes. To avoid the spectrum effect and the risk of multiple testing, we performed comparisons of diagnostic accuracy of six noninvasive indexes by Obuchowski method. The results of Obuchowski measure were in agreement with that of Delong test. Third, hepatic steatosis in the present study was diagnosed by liver biopsy, which is considered the gold standard for assessing hepatic steatosis. Fourth, the present study was performed in patients with CHB, so SIHBV is more suitable for detection of hepatic steatosis in patients with HBV infection.

There were two limitations in the present study. The first limitation was that the sample size of the present study was relatively small, which might affect reliability of the results. The second limitation of this study was that all subjects were patients in our hospital, which might reduce the representative of the study population. To further validate the diagnostic accuracy and clinical utility of SIHBV for hepatic steatosis in patients with HBV infection, further studies should base on a large scale and multi-center population.

In summary, for prediction of hepatic steatosis in patients with HBV infection, SIHBV has a better diagnostic accuracy, which is significantly superior to US, FLI, LAP, HSI, FLD, and Korean Score. SIHBV need further external validation in large population before it was used for prediction of hepatic steatosis in patients with HBV infection.

## Additional Information

**How to cite this article**: Zhang, Z. *et al*. Diagnostic accuracy and clinical utility of a new noninvasive index for hepatic steatosis in patients with hepatitis B virus infection. *Sci. Rep.* **6**, 32875; doi: 10.1038/srep32875 (2016).

## Figures and Tables

**Table 1:** Characteristics of subjects in model group and validation group.

Parameters	Model group (n = 182)	Validation group (n = 182)	Test value	*P* value
Male (n,%)	148 (81.3)	150 (82.4)	0.07	0.786#
Age (years)	33.8 ± 9.1	35.0 ± 9.9	−1.246	0.213
BMI (kg/m^2^)	23.3 ± 4.2	22.6 ± 3.4	1.793	0.074
WC (cm)	72.7 ± 12.5	69.4 ± 11.6	2.616	0.009
ALT (U/L)	73 (39, 150)	71 (39, 159)	0.524	0.946*
AST (U/L)	50 (34, 84)	51 (36, 80)	0.472	0.979*
γ-GT (U/L)	55 (30, 115)	54 (30, 107)	0.891	0.405*
Log DNA (copies/ml)	4.7 ± 2.8	4.6 ± 2.8	0.277	0.782
Albumin (G/L)	43.8 ± 4.7	44.7 ± 4.5	−1.702	0.09
WBC (10^9^/l)	5.7 ± 1.6	5.7 ± 1.4	0.136	0.892
HGB (G/L)	145.0 ± 16.0	144.0 ± 15.7	0.475	0.635
PLT (10^9^/l)	183.9 ± 55.1	184.7 ± 53.8	−0.145	0.885
PT (second)	12.4 ± 1.3	12.4 ± 1.3	−0.322	0.748
SUA (mmol/l)	350.5 ± 93.2	351.7 ± 100.8	−0.119	0.906
FPG (mmol/l)	4.8 ± 0.9	4.8 ± 1.1	−0.091	0.923
TC (mmol/l)	4.5 ± 1.0	4.4 ± 1.0	0.576	0.565
TG (mmol/l)	1.3 ± 0.9	1.3 ± 0.8	−0.118	0.906
HDL (mmol/l)	1.4 ± 0.4	1.3 ± 0.4	1.922	0.055
LDL (mmol/l)	2.4 ± 0.7	2.4 ± 0.8	−0.233	0.816
Inflammation Grade 1 (n,%)	8 (4.4)	10 (5.5)	2.18	0.535*
Inflammation Grade 2 (n,%)	80 (44.0)	92 (50.5)		
Inflammation Grade 3 (n,%)	69 (37.9)	59 (32.4)		
Inflammation Grade 4 (n,%)	25 (13.7)	21 (11.5)		
Fibrosis Stage 1 (n,%)	27 (14.8)	28 (15.4)	2.95	0.399*
Fibrosis Stage 2 (n,%)	72 (39.6)	69 (37.9)		
Fibrosis Stage 3 (n,%)	40 (22.0)	52 (28.6)		
Fibrosis Stage 4 (n,%)	43 (23.6)	33 (18.1)		
Steatosis grade0 (n,%)	123 (67.6)	123 (67.6)	0.56	0.906*
Steatosis grade1 (n,%)	44 (24.2)	47 (25.8)		
Steatosis grade2 (n,%)	9 (4.9)	8 (4.4)		
Steatosis grade3 (n,%)	6 (3.3)	4 (2.2)		
Fatty liver diagnosed by US (n,%)	30 (16.5)	34 (18.7)	0.3	0.582#
Antiviral therapy (n,%)	40 (22.0)	32 (17.6)	1.11	0.293#
HBeAg positive (n,%)	124 (68.1)	114 (62.6)	1.21	0.271#
HBV DNA positive (n,%)	143 (78.6)	141 (77.5)	0.06	0.80#

^*^Kruskal-Wallis H test.

^#^Chi-square test. Hepatic steatosis were diagnosed by liver biopsy.

SUA, Serum uric acid; FPG, Fasting plasma glucose; TC, Total cholesterol; TG, Triglyceride; HDL, high-density lipoprotein cholestero; LDL, low-density lipoprotein cholestero; ALT, Alanine aminotransferase; AST, Aspartate aminotransferase; γ-GT, γ-glutamyl transferase; BMI, body mass index; WC, waist circumference; WBC, white blood cell; PLT, blood platelet; HGB, hemoglobin; PT, Prothrombin time. Continuous data were expressed as mean ± SD or median (uartile range) depending on the normality of the data. Categorical variables were expressed as proportions.

**Table 2:** Diagnostic value of variables for predicting hepatic steatosis by logistic regression analysis.

Parameters	Univariate logistic regression analysis			95% CI		Multivariate logistic regression analysis*			95% CI	
	OR	*P* value	B	Lower	Upper	OR	*P* value	B	Lower	Upper
Male	2.730	0.003	1.004	1.412	5.277					
Age (years)	1.031	0.007	0.003	1.008	1.053	1.033	0.037	0.033	1.002	1.065
BMI (kg/m^2^)	1.573	0.001	0.453	1.431	1.728	1.511	0.001	0.413	1.357	1.682
WC (cm)	1.064	0.001	0.062	1.042	1.087					
ALT (U/L)	0.997	0.020	−0.003	0.995	1.0					
AST (U/L)	0.992	0.001	−0.008	0.987	0.997					
γ-GT (U/L)	1.001	0.281	0.001	0.999	1.004					
Log DNA (copies/ml)	0.875	0.001	−0.134	0.811	0.944					
Albumin (G/L)	1.147	0.001	0.137	1.088	1.210					
WBC (10^9^/l)	1.414	0.001	0.347	1.217	1.644					
HGB (G/L)	1.060	0.001	0.058	1.041	1.079	1.047	0.001	0.046	1.023	1.072
PLT (10^9^/l)	1.002	0.375	0.002	0.998	1.006					
PT (second)	0.509	0.001	−0.675	0.402	0.645					
SUA (mmol/l)	1.006	0.001	0.006	1.004	1.009	1.006	0.001	0.005	1.002	1.009
FPG (mmol/l)	1.311	0.012	0.271	1.061	1.619					
TC (mmol/l)	1.805	0.001	0.591	1.429	2.280					
TG (mmol/l)	2.224	0.001	0.799	1.575	3.140	1.610	0.013	0.476	1.104	2.348
HDL (mmol/l)	0.562	0.036	−0.577	0.328	0.962					
LDL (mmol/l)	2.418	0.001	0.883	1.772	3.302					
constant								−21.094		

^*^OR, odds ratio; B, partial regression coefficient; CI, confidence interval.

**Table 3:** Comparisons of the diagnostic accuracy between US and SIHBV for fatty liver.

Steatosis percentage	Number	Diagnosis by US		Diagnosis by SIHBV		Chi square test	*P* value
	n	present	absent	present	absent		
0–5.0%	246	8 (3.3%)	238 (96.7%)	16 (6.5%)	230 (93.5%)	2.8	0.094
5.0–9.9%	55	16 (29.1%)	39 (70.9%)	35 (63.6%)	20 (36.4%)	13.08	0.001
10.0–19.9%	24	11 (45.8%)	13 (54.2%)	19 (79.2%)	5 (20.8%)	5.57	0.018
20.0–32.9%	12	7 (58.3%)	5 (41.7%)	10 (83.3%)	2 (16.7%)	1.74	0.187
33.0–100.0%	27	22 (81.5%)	5 (18.4%)	22 (81.5%)	5 (18.4%)	0	1
Total correct rate	364	294 (80.8%)		316 (86.8%)		4.89	0.027

Note: Diagnosis of fatty liver by SIHBV was defined as predictive probability value ≥0.5.
